# Two is better than one: longitudinal detection and volumetric evaluation of brain metastases after Stereotactic Radiosurgery with a deep learning pipeline

**DOI:** 10.1007/s11060-024-04580-y

**Published:** 2024-02-01

**Authors:** Yonny Hammer, Wenad Najjar, Lea Kahanov, Leo Joskowicz, Yigal Shoshan

**Affiliations:** 1 School of Computer Science and Engineering, The Hebrew University of Jerusalem, Edmond. J. Safra Campus, Givat Ram, 9190401 Jerusalem, Israel; 2 Department of Neurosurgery, Hadassah Hebrew University Medical Center, Jerusalem, Israel; 3 Edmond and Lily Safra Center for Brain Sciences, The Hebrew University of Jerusalem, Jerusalem, Israel

**Keywords:** Stereotactic radiosurgery evaluation, Brain metastases detection and segmentation, Volumetric brain metastases assessment, Longitudinal evaluation

## Abstract

**Purpose:**

Close MRI surveillance of patients with brain metastases following Stereotactic Radiosurgery (SRS) treatment is essential for assessing treatment response and the current disease status in the brain. This follow-up necessitates the comparison of target lesion sizes in pre- (prior) and post-SRS treatment (current) T1W-Gad MRI scans. Our aim was to evaluate SimU-Net, a novel deep-learning model for the detection and volumetric analysis of brain metastases and their temporal changes in paired prior and current scans.

**Methods:**

SimU-Net is a simultaneous multi-channel 3D U-Net model trained on pairs of registered prior and current scans of a patient. We evaluated its performance on 271 pairs of T1W-Gad MRI scans from 226 patients who underwent SRS. An expert oncological neurosurgeon manually delineated 1,889 brain metastases in all the MRI scans (1,368 with diameters > 5 mm, 834 > 10 mm). The SimU-Net model was trained/validated on 205 pairs from 169 patients (1,360 metastases) and tested on 66 pairs from 57 patients (529 metastases). The results were then compared to the ground truth delineations.

**Results:**

SimU-Net yielded a mean (std) detection precision and recall of 1.00±0.00 and 0.99±0.06 for metastases > 10 mm, 0.90±0.22 and 0.97±0.12 for metastases > 5 mm of, and 0.76±0.27 and 0.94±0.16 for metastases of all sizes. It improves lesion detection precision by 8% for all metastases sizes and by 12.5% for metastases < 10 mm with respect to standalone 3D U-Net. The segmentation Dice scores were 0.90±0.10, 0.89±0.10 and 0.89±0.10 for the above metastases sizes, all above the observer variability of 0.80±0.13.

**Conclusion:**

Automated detection and volumetric quantification of brain metastases following SRS have the potential to enhance the assessment of treatment response and alleviate the clinician workload.

**Supplementary Information:**

The online version contains supplementary material available at 10.1007/s11060-024-04580-y.

## Introduction

Brain metastases are the most common type of brain tumors, affecting 10–40% of patients with solid tumors during their clinical course. Their prevalence is ~ 10 times higher than that of primary malignant brain tumors. About half of the patients with clinical and radiological evidence of cerebral metastases present multiple lesions during the course of their disease [1, 2].

Close MRI surveillance of patients with brain metastases following Stereotactic Radiosurgery (SRS) treatment is essential for assessing treatment response, current disease status, and the emergence of new metastases. This follow-up necessitates the comparison of irradiated lesion sizes and characteristics in pre-treatment (baseline, prior) and post-treatment (follow-up, current) T1W-Gad MRI scans acquired every 2–3 months. Longitudinal radiological tracking of cerebral metastatic patients usually involves numerous metastases, thus posing significant challenges. It is prone to errors, might be affected by inter and intra-observer variability, and may be a time-consuming task for neurosurgeons, oncologists, and radiologists.

Recently, deep learning models have demonstrated significant promise in medical image analysis, excelling in tasks such as detection and segmentation. Stereotactic radiosurgery for brain metastases relies on accurate tumor detection and precise lesion volume, emerges as a promising candidate for Artificial Intelligence (AI) model development [3–8].

In a recent paper [9], we described a novel automatic pipeline for the simultaneous detection of liver lesions and their changes in longitudinal contrast-enhanced CT liver scans. This pipeline includes SimU-Net, a simultaneous multi-channel 3D U-Net model trained on pairs of registered scans of each patient. The model identifies liver lesions and their changes based on the differences in the appearance between the lesion and the healthy tissue. It matches and classifies the lesion changes and produces a comprehensive report of temporal lesion changes.

**Fig. 1:**
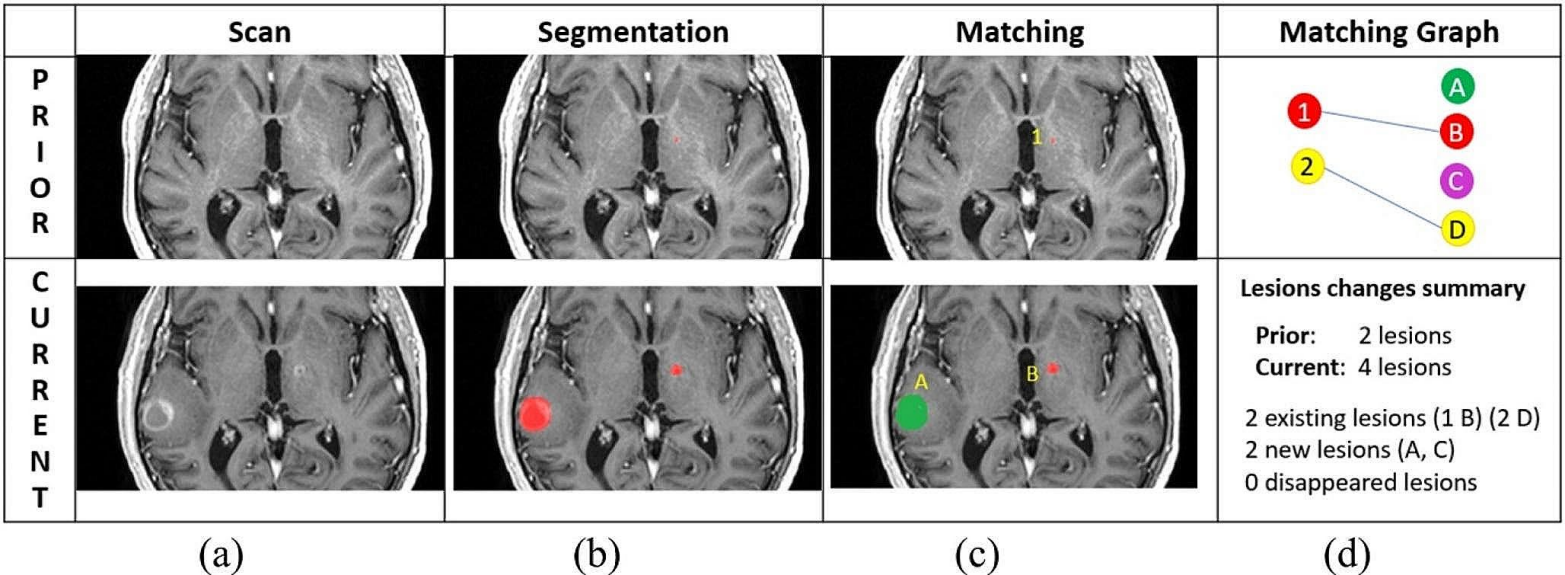
Illustration of the analysis of temporal changes of brain metastases of a patient before and after SRS: (**a**) representative registered slices of prior (top) and current (bottom) T1W-Gad scans; (**b**) annotation of two brain metastases (red) in the prior scan slice (top) and one in the current scan slice (bottom); one additional brain metastases in the prior scan and two in the current scans appear in different slices are not shown; (**c**) matching of the detected brain metastases in the prior scan (top, numbers) and in the current scan (bottom, letters) for the brain metastases appearing in the slices: metastasis 1 (red) in the prior scan slice and metastases A (green) and B (red) in the current slice scan. Brain metastases 2 (prior scan) and C and D (current scan) appear different slices are not shown; (**d**) lesion matching graph for the entire prior and current scans (top): nodes correspond to brain metastases in the prior (left) and current (right) scans; edges correspond to lesion matchings; the matching of 1 and B is seen in the prior and current scan slices, while the matching of 2 and D appear on other slices. Summary of lesion (brain metastases) changes (bottom): number of lesions in each scan and of each type of lesion changes– existing (persistent), new, disappeared (numbers and letters indicate matchings). Volumetric measurements are also computed but were omitted here

In the current study, we have extended the simultaneous analysis pipeline SimU-Net for the detection of cerebral metastases and their temporal changes in longitudinal brain T1W-Gad scans. Our primary hypothesis was that a deep neural network trained on two consecutive scans yields superior accuracy and recall than the same state-of-the-art single, standalone 3D nnU-Net model trained on the same scans. Our secondary hypothesis was that the pipeline can successfully match cerebral metastases and correctly classify their temporal changes (Fig. 1).

The aim of the current study was to evaluate the performance of the SimU-Net pipeline for pairs of pre- and post SRS T1W-Gad scans for the detection and segmentation of cerebral metastases and for their matching and classification of lesion changes.

## Materials and methods

**Fig. 2:**
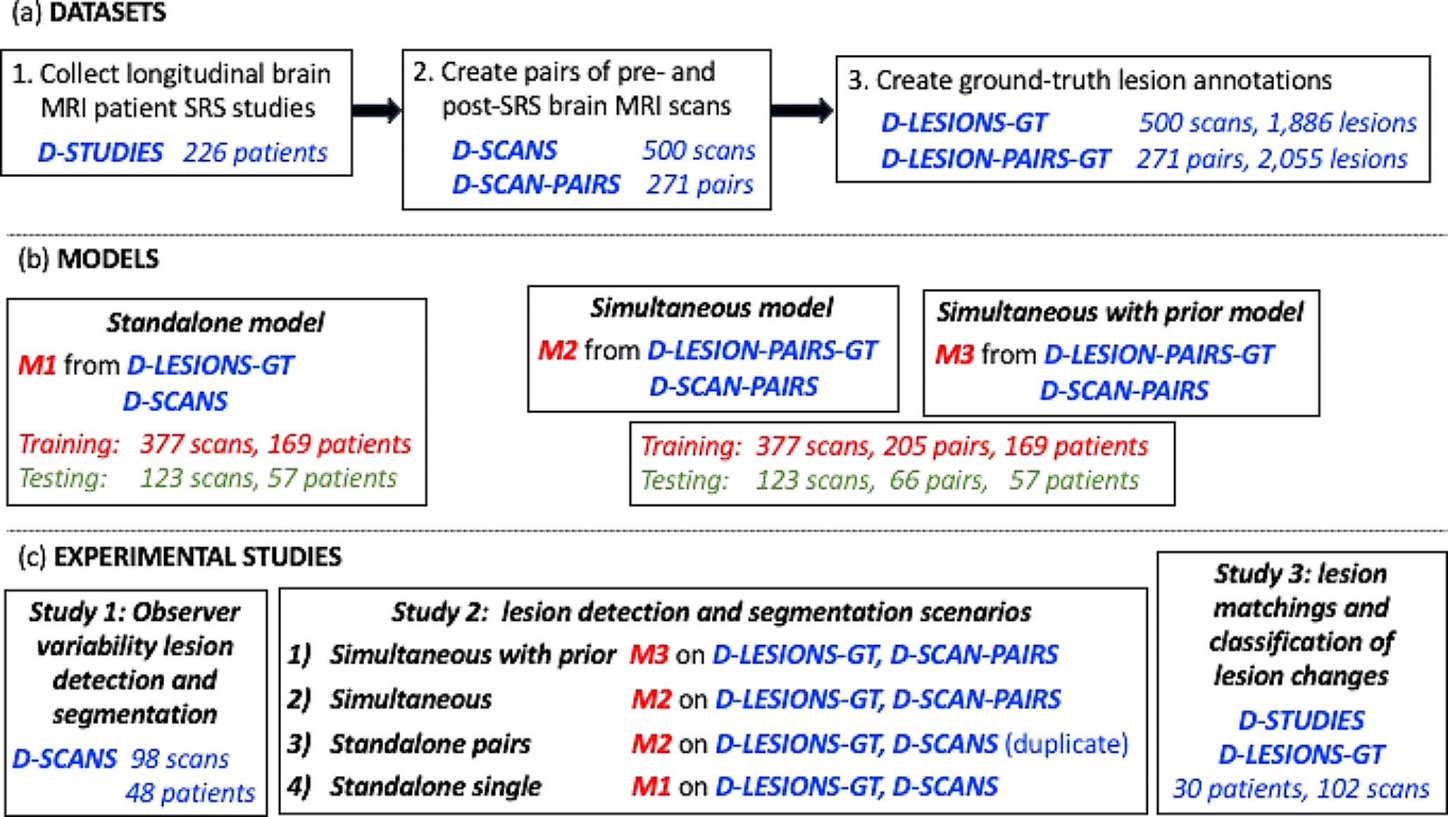
(**a**) flowchart of the steps of the creation of the three scans datasets ***D-STUDIES, D-SCANS, D-SCAN-PAIRS*** and the two manual brain metastases (lesions) annotation datasets ***D-LESIONS-GT*** and ***D-LESION-PAIRS-GT***; (**b**) three deep learning models ***M1, M2, M3*** and their training and testing datasets; (**c**) experimental studies with the models and the testing datasets used to conduct them. The number of patients, scans, pairs of scans and brain metastases are listed

Figure 2 summarizes the dataset creation process, the deep learning models, and the experimental studies and their characteristics.

### Patient studies

Brain MRI studies of patients with metastatic disease who underwent SRS were retrospectively obtained from Hadassah Ein Kerem University Medical Center (Jerusalem, Israel) by the clinician co-authors. The studies, each with at least two scans, were acquired in 2011-22. Studies of patients with non-metastatic cancer or who underwent surgery were excluded. Patients’ details were anonymized; informed consent was waived and IRB approval was obtained (0338 − 20 HMO). The scans were acquired on three scanners: GE SignaTM Voyager (Chicago, USA) Siemens Magnetom Avantofit (Munich, Germany) and Philips Ingenia (Amsterdam, Netherlands). The scans voxel sizes were 0.2–1.2 × 0.2–1.2 × 0.7-4.4mm^3^.

LINAC-based SRS and FSR treatment was delivered by the LINAC-based platform available at the time (2005-15 Varian DBX with Brainlab M3 and 2016-20Truebeam Novalis STx with ExacTrac X-Ray). Patients were assigned to receive either Fractionated Stereotactic Radiotherapy (FSR) or Single Fraction Stereotactic Radiosurgery (SRS) based on a multidisciplinary discussion in adherence to consensus guidelines. For non-brain stem metastases, a single-shot treatment of 18–24 Gy (RTOG 90 − 05) was prescribed, while metastases whose volume exceeded 6 cc were treated with a marginal dose of 27 Gy administered in three daily fractions. Before 2016, single-fraction SRS treatment was performed with rigid stereotactic frame head fixation; FSR treatments utilized a rigid thermoplastic face-specific mask. From 2016 on, both SRS and FSR patients underwent treatment with a rigid thermoplastic face-specific mask. The imaging protocol included high-definition CT scans followed by Axial 3D T1W-GaD MRI with 0.5 mm slice thickness sequences. The fusion of the data sets was performed through treatment planning, delineating tumor volume and organs at risk. Treatment planning involved dynamic conformal arc therapy or Intensity Modulated Radiotherapy (IMRT) and received approval from the treating physician. Patients who had undergone a recent craniotomy for the resection of large cerebral metastases were excluded from the study. This exclusion was based on the challenges posed by multiple post-surgical changes, metal artifacts, and the inherent difficulties in accurately delineating the tumor bed and distinguishing between residual tumor and post-operative changes.

### Scans datasets

Three datasets of scans were created (Fig. 2a). Dataset ***D-STUDIES*** included 226 patient studies of 110 males and 116 females, mean age 63, range 23–89 years (step 1). The primary tumor sources (number of patients) were lungs (116), breast (49), gastrointestinal tract (17), melanoma (14), renal (11), other (19), with a mean of 4.6 (std = 3.2, range = 0.7–18.1) months between scans (no demographic information for the last three categories was available).

Dataset ***D-SCANS*** consisted of 500 T1W-Gad scans from the 226 patient studies (step 2).

Dataset ***D-SCAN-PAIRS*** consisted of pairs of time-ordered pre-SRS and post-SRS scans of the same patient created from dataset ***D-SCANS*** (step 2). The pairs were created by pairing the pre-SRS scan with one of the follow-up post-SRS scans. This resulted in a total of 271 pairs from the 500 brain MRI scans.

### Manual annotations datasets

Two brain metastases (lesions) annotations datasets were created as follows (Fig. 2a, step 3). Dataset ***D-LESIONS-GT*** consisted of ground-truth annotations of brain metastases created from the patient scans in ***D-SCANS*** as follows. First, manual annotations for 1,571 lesions in 439 scans were created using ITK-SNAP [10] by the two senior neurosurgeons’ co-authors (> 30 and > 10 years of experience). Second, 318 lesions in the remaining 61 scans were created by having the radiologists correct the segmentations computed by SimU-Net. This resulted in a total of 1,889 lesions, of which 1,368 are > 5 mm in diameter (0.07 cc) and 834 are > 10 mm in diameter (0.52 cc), with a mean number of lesions/scan of 3.6 ± 5.11 and mean lesion diameter of 10.84 mm (0.66 cc).

Dataset ***D-LESION-PAIRS-GT*** consisted of the corresponding pairs of lesion annotations in ***D-SCAN-PAIRS***. It included a total of 2,055 lesions (more than 1,889 because of pre-SRS scan repetitions in ***D-SCAN-PAIRS***), of which 1,748 are > 5 mm in diameter (0.07 cc) and 1,065 are > 10 mm in diameter (0.52 cc), with a mean number of lesions/scan of 3.6 ± 4.9 and a mean lesion diameter of 10.69 mm (0.64 cc).

### Method

We extended the pipeline described in [9] for metastatic brain lesions. The input is a pair of prior and current brain MRI scans, and, when available, the prior scan lesion segmentations. The output is the analysis of lesion changes consisting of the lesion segmentation in the scans and the report of the lesion changes (Fig. 1d).

The pipeline uses SimU-Net, a simultaneous multi-channel model trained on pairs of registered scans of each patient. The SimU-Net is a modified 3D U-Net [11] in which the encoding path single-channel input layer is replaced by a multiple-input layer. The two-channel SimU-Net inputs matching pairs of 3D patches from the registered prior and current scans. The three-channel SimU-Net inputs in addition the lesions segmentation masks in the prior scan.

**Fig. 3:**
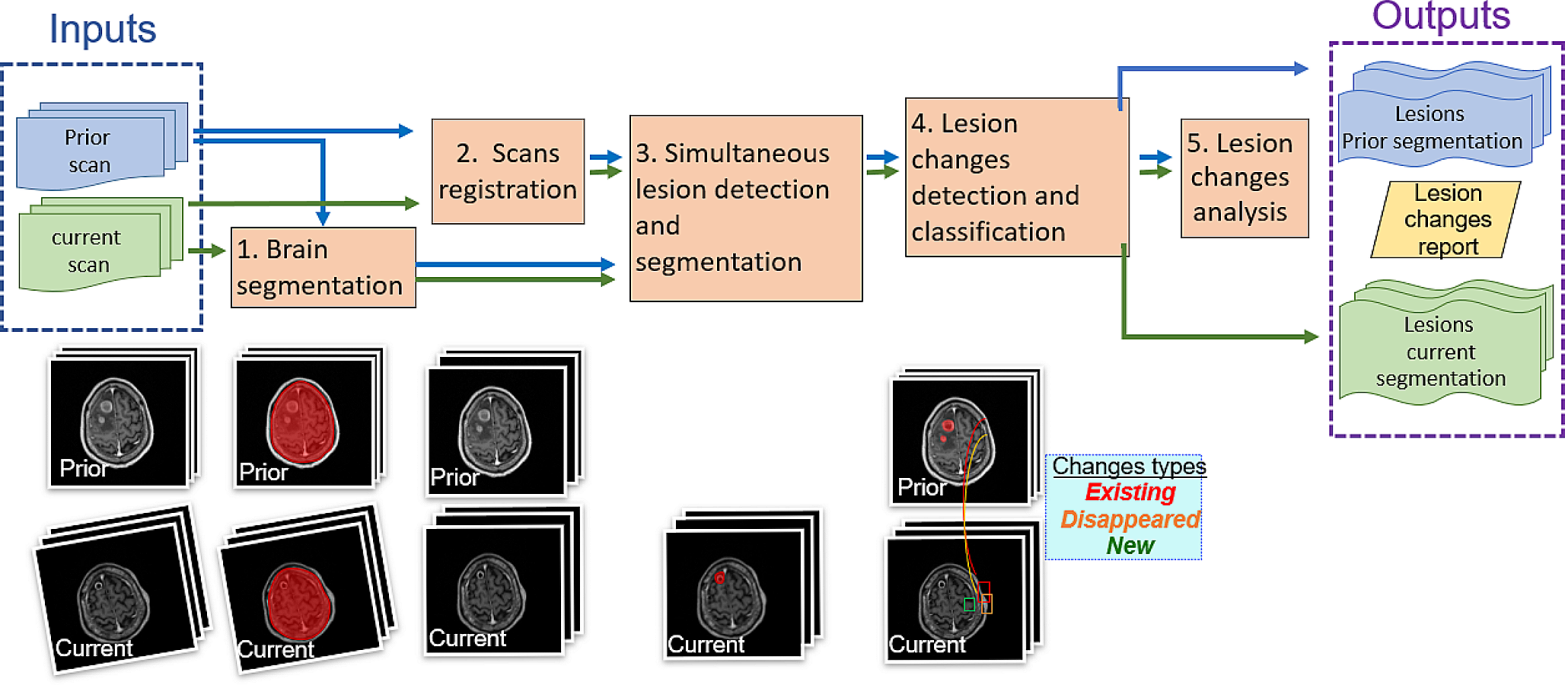
Flow diagram of the SimU-Net pipeline for the analysis of brain metastases (lesions) and their temporal in pairs of pre- and post-SRS brain T1W-Gad MRI scans. The inputs are the prior and the current MRI scans of a patient. The outputs are the lesion segmentations in each scan and the lesion changes report. The steps are: (1) brain segmentation in the prior and current scans; (2) registration of the prior scan to the current scan; (3) simultaneous brain lesion detection and segmentation in the prior and current scans with SimU-Net; (4) detection and classification of lesion changes; (5) quantification of lesions and lesion changes

The pipeline consists of five steps (Fig. 3): (1) brain segmentation in each of the prior and the current scans with a deep learning brain classifier model; (2) registration of the prior and the current scans and of their brain segmentations; (3) simultaneous lesion detection and segmentation in the current and prior brain with SimU-Net; (4) detection and classification of lesion changes with a graph-based lesion matching method; (5) quantification of lesions and lesion changes.

We briefly describe next the adaptations made to the metastatic liver lesions pipeline for the brain MRI scans next.

For brain segmentation (Step 1), we remove the skull using the SynthStrip model described in [12]. Accurate brain segmentation is not necessary, as a brain region of interest is sufficient to filter out false positive lesions outside the brain. For the registration of the prior and current scans, we perform rigid registration with FreeSurfer [13] since deformable registration is not needed. For brain metastases detection and segmentation (Step 3), we use a SimU-Net model trained on pairs of brain scans and their manual delineations. Steps 4 and 5 are generic and are thus performed with the method described in [14].

### Deep learning models

Three deep learning models for brain metastases detection and segmentation were built (Fig. 2b): (1) ***M1***, a single-channel 3D nnU-Net model trained on individual scans [15]; (2) ***M2***, a two-channel SimU-Net model trained on registered pairs of prior and current scans; (3) ***M3***, a three-channel SimU-Net model trained on pairs of registered pairs of current and prior scans and prior lesion segmentations. To allow direct comparison of their performance, the training/validation and test sets were the exact same ones for the three models. Model ***M1*** was trained/validated on 377 scans (169 patients, 1,360 metastases) and tested on 123 scans (57 patients, 529 metastases) from ***D-SCANS*** and ***D-LESIONS-GT***. Models ***M2*** and ***M3*** were trained/validated on 205 pairs (same 169 patients and scans than for ***M1***) and tested on 66 pairs (same 57 patients and scans than for ***M1***) from ***D-SCAN-PAIRS*** and ***D-LESION-PAIRS-GT***. The training, validation and test sets were chosen so that their distribution for each lesion diameter class reflects that of the entire dataset.

For the visual evaluation of the lesion detection, segmentation, and matching, we developed a viewer that allows the simultaneous viewing of synchronized scan slices. Fig. S2 in the Supplemental Material shows a screenshot of the viewer, which proved to be of great use for the experimental studies.

### Experimental studies

We conducted three experimental studies as follows (Fig. 2c).

#### Study 1

Quantifies the observer variability of the detection and segmentation of the brain metastases by two expert neurosurgeons [16]. Each clinician independently annotated the brain metastases on 98 scans from 48 patients from ***D-SCANS***. The annotations were compared to establish the relative detection precision and recall and lesion segmentation Dice score. The clinicians examined the discrepancies and established consensus.

#### Study 2

Quantifies the performance of the SimU-Net pipeline on ***D-LESIONS***-***GT*** in four scenarios: (1) ***Simultaneous with prior***: inference on current and prior scans and prior lesion segmentation with ***M3***; (2) ***Simultaneous***: inference on a pair of registered prior and current scans with ***M2***; (3) ***Standalone pairs***: inference on a single scan with ***M2*** performed by using the same scan in both input channels (duplicate); (4) ***Standalone single***: inference on a single scan with ***M1***–– the reference scenario for comparison with the state-of-the-art.

#### Study 3

Quantifies the performance of the lesion matchings and the classification of lesion changes of the prior and current brain segmentations on a subset of ***D-STUDIES*** and their corresponding lesion annotations in ***D-LESIONS-GT***. Lesion matchings and classification of lesion changes were created by automatically registering the scans, computing an initial lesion matching with the method in [9] and subsequently having the senior neurosurgeon review and correct them. The set consisted of 30 patient studies (14 males, 16 females, mean age of 62, range 34–77) with at least three scans from different timepoints per patient. In total, there were 102 scans with a mean 3.4 ± 0.5 scans/patient (maximum 5 scans/patient) and mean time of 102.1 ± 93.6 days between consecutive scans It included a total of 266 lesions, of which 142 are > 5 mm and 38 are > 10 mm. The mean number of lesions/scan was 7.79 ± 6.34 with a mean lesion diameter of 6.27 (0.13 cc). Of the 266 lesions, 128 are existing lesions, 66 are lesions that disappeared, and 72 are new lesions.

### Statistical analysis

Lesion detection was evaluated with the standard precision and recall and their standard deviation. Lesion segmentations were evaluated with the Dice score (Dice) and the Average Symmetric Surface Distance (ASSD). Independent t-test two-tails comparison with *p* < 0.01 set for statistical significance was performed.

The lesion matching and classification of lesion changes were evaluated on the lesions matching graph with the precision and recall of two metrics: (1) matching lesions (edges): a True Positive edge is present in both the ground truth and the computed graph; a False Positive edge is a computed edge that is not present in the ground truth graph; a False Negative edge is present in the ground truth graph and is missing in the computed graph; (2) Classification of lesion changes: comparison between the computed and the ground truth classes of lesion changes: new, existing and disappeared.

## Results

### Study 1

Table S1 in the Supplemental Material summarizes the observer variability results. For lesions > 5 mm, the mean precision is 0.88 ± 0.24, the mean recall is 0.96 ± 0.12, the mean Dice is 0.82 ± 0.12 and the mean ASSD is 0.65 ± 0.78. For lesions > 10 mm, the mean precision is 0.95 ± 0.19, the mean recall is 0.98 ± 0.12, the mean Dice is 0.85 ± 0.10 and the mean ASSD is 0.66 ± 0.76. For lesions of all sizes, the mean precision is 0.92 ± 0.15, the mean recall is 0.85 ± 0.23, the mean Dice is 0.80 ± 0.13 and the mean ASSD is 0.62 ± 0.75. This establishes the consensus ground truth between two clinicians and sets the desired goal for the automatic analysis.

### Study 2

Table 1 summarizes the results. For lesion detection, all scenarios yield a perfect recall and perfect precision (1.00 ± 0.00) for lesions > 10 mm. For all scenarios and all sizes, the Recall was not statistically significantly. However, the Precision of the Simultaneous scenarios was statistically significant.

For lesions > 5 mm, all scenarios yield high, very similar mean recall range of 0.95–0.96 with a std range of 0.13–0.14; the ***Simultaneous without prior*** and ***Standalone pairs*** scenarios yield the highest mean precision range of 0.92–0.93 with a std range of 0.18–0.19 vs. mean precision range of 0.86–0.89 with a std range of 0.24–0.28. For all lesions, the ***Standalone*** scenarios yield slightly better mean recall range of 0.82–0.83 with std range of 0.28–0.29 vs. mean recall of 0.80 with std range of 0.28–0.31; the ***Simultaneous with prior*** scenario yield the highest mean precision of 0.83 ± 0.24 vs. mean precision range of 0.75–0.78 with a mean std range of 0.26–0.28. This constitutes a statistically significant improvement in the mean lesion detection precision of 8% for all lesion sizes (0.83–0.765)/0.83 × 100), and 12.5% for lesions < 10 mm ((0.72–0.63)/0.72 × 100).

For lesion segmentation, the Dice score and ASSD are above the observer variability for all scenarios and lesion sizes, with a larger std in some cases: 0.80–0.90 ± 0.10–0.21 computed vs. 0.80–0.85 ± 0.10–0.13 manual and 0.27–0.62 ± 0.35-1.27 mm computed vs. 0.51–0.66 ± 0.56–0.76 manual, respectively. The ***Simultaneous without prior*** scenario yields the best Dice scores for all lesion sizes, 0.83–0.90 ± 0.10–0.22. For the ASSD, the results are mixed.

**Fig. 4:**
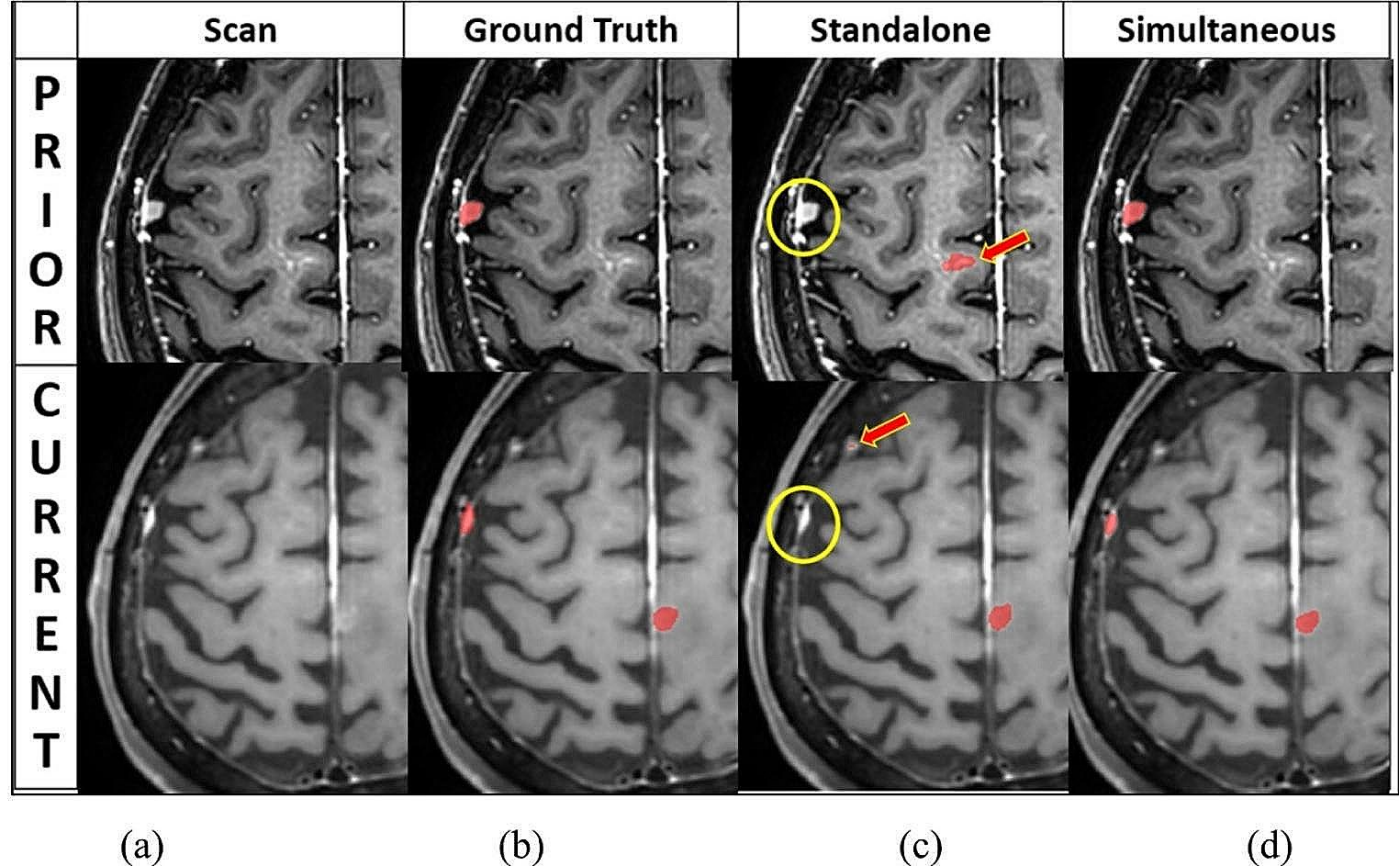
Illustration of the performance of the ***Simultaneous with prior*** and the ***Standalone*** scenarios: (**a**) Representative matching slices of prior (top) and current (bottom) brain MRI scans of a patient acquired three months apart; (**b**) Ground truth lesions’ segmentation (red) of prior (top) and current (bottom) scans; (**c**) Standalone model results: lesions are not detected (yellow circle) and over-detected (red arrow) in prior and current scans; (**d**) Simultaneous with prior model results: the lesion is correctly detected and accurately segmented in both the prior (top) and current (bottom) scans

Figure 4 illustrates the advantage of the ***Simultaneous without prior*** scenario over the ***Standalone*** scenario. It demonstrates how more information yields better detection and segmentation results.

### Study 3

The SimU-Net pipeline achieves perfect results on lesion matching and classification of lesion changes: the precision and recall are 1.0 ± 0.0 on all 266 lesions.

**Table 1:** Results of the testing set detection and segmentation of brain metastases (lesions) with diameters > 10 mm, > 5 mm, < 10 mm and all sizes in four scenarios: ***Simultaneous with prior***, ***Simultaneous***, ***Standalone pairs*** and ***Standalone***. Listed are the mean (std) lesion detection precision and recall and the mean (std) lesion segmentation Dice and ASSD. Boldface numbers indicate best per-class results. The number of lesions in each category is indicated in parentheses

LESION DIAMETER (# of lesions)	SCENARIO	LESION DETECTION		LESION SEGMENTATION	
		Precision	Recall	Dice	ASSD
**> 10 mm** (167)	***Simultaneous with prior***	**1.00** (0.00)	**1.00** (0.00)	0.87 (0.14)	0.62 (0.88)
	***Simultaneous***	**1.00** (0.00)	**1.00** (0.00)	**0.90** (0.10)	0.48 (0.80)
	***Standalone*** ***pairs***	**1.00** (0.00)	**1.00** (0.00)	0.89 (0.15)	0.63 (1.06)
	***Standalone single***	**1.00** (0.00)	**1.00** (0.00)	**0.90** (0.11)	**0.46** (0.60)
**> 5 mm** (204)	***Simultaneous with prior***	0.89 (0.24)	**0.96** (0.13)	0.85 (0.17)	0.50 (0.67)
	***Simultaneous***	0.92 (0.19)	0.95 (0.14)	**0.88** (0.15)	0.41 (0.60)
	***Standalone*** ***pairs***	**0.93** (0.18)	**0.96** (0.13)	0.87 (0.18)	0.50 (0.82)
	***Standalone single***	0.86 (0.28)	0.95 (0.13)	0.87 (0.15)	**0.39** (0.48)
**< 10 mm** (158)	***Simultaneous with prior***	**0.72** (0.34)	0.75 (0.34)	0.80 (0.15)	0.30 (0.35)
	***Simultaneous***	0.68 (0.36)	0.77 (0.34)	**0.83** (0.16)	**0.27** (0.43)
	***Standalone*** ***pairs***	0.67 (0.36)	**0.81** (0.33)	0.82 (0.22)	0.43 (1.27)
	***Standalone single***	0.63 (0.37)	0.80 (0.32)	0.81 (0.17)	0.28 (0.42)
**All** (529)	***Simultaneous with prior***	**0.83** (0.24)	0.80 (0.28)	0.83 (0.17)	0.46 (0.59)
	***Simultaneous***	0.77 (0.28)	0.80 (0.31)	**0.87** (0.14)	0.36 (0.50)
	***Standalone*** ***pairs***	0.78 (0.28)	0.82 (0.29)	0.85 (0.19)	0.50 (0.83)
	***Standalone single***	0.75 (0.26)	**0.83** (0.28)	0.86 (0.16)	**0.35** (0.44)

## Discussion

Our studies show that the simultaneous detection and segmentation of brain metastases with a SimU-Net outperforms a standard standalone 3D U-Net trained on the same dataset. SimU-Net also improves lesion detection in a single scan when the deep learning model was trained on pairs of scan annotations. When SimU-Net also incorporates the prior lesion segmentations, it further improves lesion detection, yielding a statistically significant improvement in the precision by 8% for all lesion sizes, and 12.5% for lesions < 10 mm. These results establish the state-of-the-art performance and are within the manual delineation observer variability.

The experimental results confirm our hypothesis that the accuracy and robustness of the detection and segmentation of brain lesions in the current scan improves as more information about the prior scan and prior lesion segmentation is added. The simultaneous deep learning networks leverage the radiological information in the same brain location from two time points and thus increase the accuracy of the voxel-level classification. It decreases the number of false negative lesion detections and improves lesion segmentation accuracy. Our results also show that a deep learning model trained on pairs of registered prior and current scans outperforms a model trained on individual scans with the exact same training and validation scans.

The precision and recall quantify the performance of the method on wrongly identified brain metastases (false positives) and missed brain metastases (false negatives). In a clinical setting, wrongly identified lesions are easier deal with, as they can be inspected by an expert on few slices and can be eliminated with a single mouse click. However, missed lesions require the inspection of the entire scan. SimU-Net has an excellent recall of 0.96 ± 0.13 and precision of 0.93 ± 0.18 for lesions > 5 mm, which are the lesions that are reported.

The method for lesion matching and for classification of lesion changes achieve a perfect score. The SimU-Net pipeline is an accurate, reliable and fully automatic end-to-end method for the comprehensive analysis of longitudinal changes of brain metastases in MRI scans.

Previously published studies used methods based on Convolutional Neural Networks (CNN). In particular, models based on the U-Net [11] are commonly used for detection and segmentation of medical images [17]. Ozkara et al. [18] present a review of recent methods for the detection and segmentation of brain lesions in individual MRI scans. Methods for the reduction of false positive lesions have been proposed [19, 20]. Methods that perform longitudinal analysis by individual, standalone detection and segmentation in each scan and then compare the results have been developed for specific primary cancer [21]. Other methods incorporate multimodal scans [6] and radiomics information [22], but none performs simultaneous lesion comparison and lesion matching. Cassinelli-Petersen [23] describe a method that incorporates validated prior lesion annotations for the segmentation of existing lesions; however, it does not detect new lesions and does not provide a comprehensive analysis of brain lesion changes as shown in Fig. 1.

The potential advantages of our automated method include the provision of the most important clinical measurements that clinicians and radiologists use to evaluate disease status: detection, classification, segmentation, and evaluation of lesion changes in longitudinal MRI scans. It may save the clinicians significant time and effort by automatically identifying and quantifying brain lesion changes and may increase the radiologists’ reading confidence by providing accurate volumetric measurements of lesions and lesion changes. Moreover, it may help identify small, subtle lesion changes that might otherwise be overlooked. Detecting small brain metastases is particularly critical for patients with low compliance and those residing in rural areas. The viewer that we developed is designed to significantly facilitate the clinician’s work when dealing with patients who come for clinic follow-up after diagnosis and treatment of brain metastases.

Our studies have several limitations. The data was collected in a single medical center and was acquired on three different scanner types. Cases of patients who underwent initial surgery for their metastasis, or had surgery between scans were excluded. The brain metastases ground truth was established by performing manual detection and delineation, which is subject to error and observer variability, especially for small lesions, and lesions with fuzzy contours.

In conclusion, employing a method that simultaneously analyzes pairs of brain MRI scans with the suggested algorithm proves to be more effective than independently assessing each study for evaluating brain metastases. The integration of the algorithm-based viewer has the potential to enhance clinical decision-making efficiency by offering precise and dependable volumetric measurements of lesions and their changes. This capability supports the evaluation of disease status, assessment of treatment efficacy, and monitoring response to therapy, contributing to significant time savings in the clinical setting.

### Electronic supplementary material

Below is the link to the electronic supplementary material.

Supplementary Material 1

## Data Availability

No datasets were generated or analysed during the current study.
