# Supplementary material for: Two is better than one: longitudinal detection and volumetric evaluation of brain metastases after Stereotactic Radiosurgery with a deep learning pipeline
Source: J Neurooncol. 2024 Feb 1;166(3):547–55. doi: 10.1007/s11060-024-04580-y (PMC10876809; doi:10.1007/s11060-024-04580-y)
Supplement: Supplementary file 1 — Supplementary Material 1 [file 11060_2024_4580_MOESM1_ESM.docx]

**Supplementary Material**

**Two is Better than One: Longitudinal Detection and Volumetric Evaluation of Brain Metastases after Stereotactic Radiosurgery with a Deep Learning Pipeline**


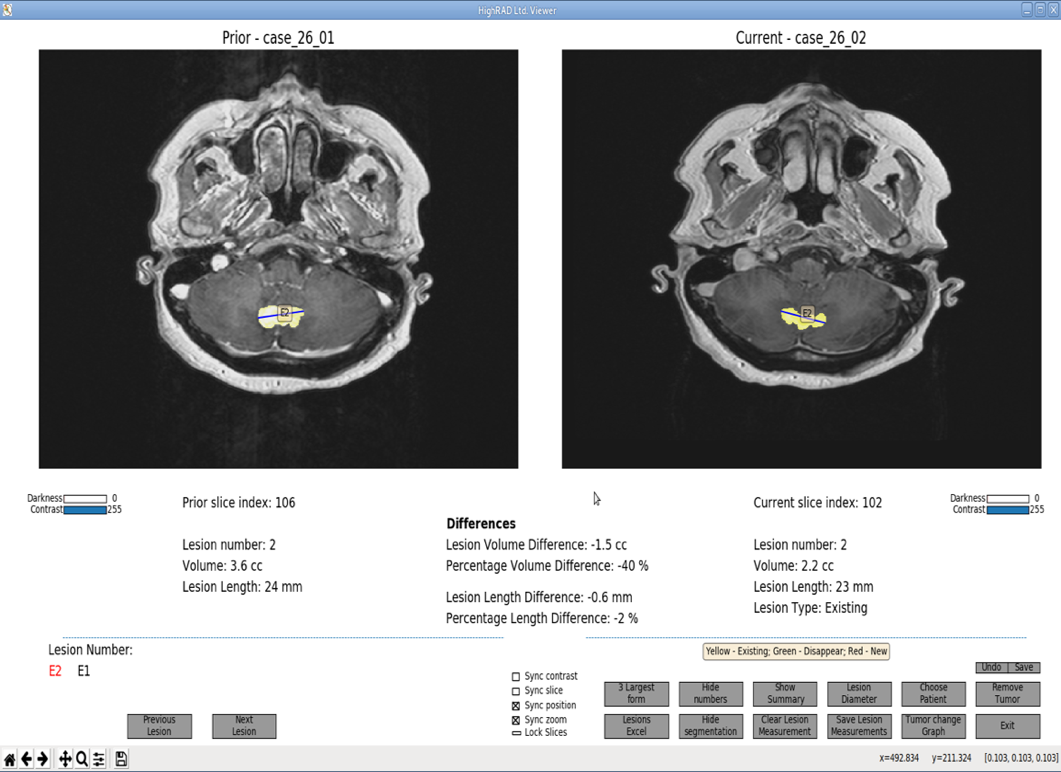


**Fig. S1**: Illustrative screen shot of the viewer for the simultaneous review of prior and current scans. Top: brain metastasis (lesion) delineation (yellow), name and diameter (blue); bottom: summary of lesion and lesion changes: number of lesions, diameter, volume and differences.

| **LESION SEGMENTATION** | | **LESION DETECTION** | | **LESION DIAMETER**  (# of lesions) |
| --- | --- | --- | --- | --- |
| **ASSD** | **Dice** | **Recall** | **Precision** |  |
| 0.66  (0.76) | 0.85  (0.10) | 0.98  (0.12) | 0.95  (0.19) | **> 10 mm**  (167) |
| 0.65  (0.78) | 0.82  (0.12) | 0.96  (0.12) | 0.88  (0.24) | **> 5 mm**  (285) |
| 0.51  (0.56) | 0.71  (0.15) | 0.80  (0.32) | 0.88  (0.26) | **< 10 mm**  (118) |
|  |  |  |  |  |
| **0.62**  **(0.75)** | **0.80**  **(0.13)** | **0.85**  **(0.23)** | **0.92**  **(0.15)** | **All**  **(343)** |

**Table S1**: Results of the observer variability of the detection and segmentation of brain metastases (lesions) by two expert neurosurgeons for lesions > 10mm, > 5mm, < 10mm, and all: mean (std) lesion detection precision and recall and mean (std) lesion segmentation Dice and ASSD. The number of lesions in each category is indicated in parentheses.
